# DNA methylation of a NF-κB binding site in the aquaporin 5 promoter impacts on mortality in sepsis

**DOI:** 10.1038/s41598-019-55051-8

**Published:** 2019-12-06

**Authors:** Katharina Rump, Matthias Unterberg, Agnes Dahlke, Hartmuth Nowak, Björn Koos, Lars Bergmann, Winfried Siffert, Simon T. Schäfer, Jürgen Peters, Michael Adamzik, Tim Rahmel

**Affiliations:** 1 Klinik für Anästhesiologie, Intensivmedizin und Schmerztherapie, Universitätsklinikum Knappschaftskrankenhaus Bochum, D-44892 Bochum, Germany; 2 Institut für Pharmakogenetik, Universität Duisburg-Essen & Universitätsklinikum Essen, D-45122 Essen, Germany; 3 Klinik für Anästhesiologie und Intensivmedizin, Universität Duisburg-Essen & Universitätsklinikum Essen, D-45122 Essen, Germany

**Keywords:** Molecular medicine, Risk factors, Sepsis

## Abstract

Altered aquaporin 5 (*AQP5*) expression in immune cells impacts on key mechanisms of inflammation and is associated with sepsis survival. Since epigenetic regulation via DNA methylation might contribute to a differential *AQP5* expression in sepsis, we tested the hypotheses that DNA methylation of the *AQP5* promotor (1) influences *AQP5* expression, (2) is associated with the 30-day survival of septic patients, and (3) alters the nuclear transcription factor NF-κB binding. *AQP5* mRNA expression was quantified by real-time PCR in whole blood samples of 135 septic patients. *In silico* computer analysis of the *AQP5* promoter (nt-567 to nt-975) revealed seven putative inflammatory transcription factor binding sites and methylation of these sites was analyzed. Electrophoretic mobility shift assays were performed to assess the binding of nuclear NF-κB to the *AQP5* promoter region nt-937. After adjustment for multiple testing, a greater methylation rate was found at cytosine site nt-937 in the *AQP5* promoter linked to NF-κB binding in non-survivors compared to survivors (p = 0.002, p_adj_ = 0.014). This was associated with greater *AQP5* mRNA expression in non-survivors (p = 0.037). Greater (≥16%) promoter methylation at nt-937 was also associated with an independently increased risk of death within 30 days (HR: 3.31; 95% CI: 1.54–6.23; p = 0.002). We detected a functionally important *AQP5* promoter cytosine site (nt-937) linked to the binding of the inflammatorily acting nuclear transcription factor NF-κB, with increased methylation in sepsis non-survivors. Thus, nt-937 APQ5 promoter methylation, presumably related to NF-κB binding, is prognostically relevant in sepsis and demonstrates that epigenetic changes impact on sepsis outcome.

## Introduction

Sepsis is a grave medical condition and its mortality remains high^1^. Thus, identification of diagnostic and therapeutic targets is a corner-stone of current research. Since the wide variability regarding sepsis outcome cannot be explained solely by patients’ comorbidities and severity of the triggering infection, some of this variability may be influenced by genetic variations that, in turn, may provide insights into relevant sepsis mechanisms.

Aquaporins seem to be a promising diagnostic and therapeutic target^2^, specifically the gene encoding aquaporin 5 (*AQP5*)^3^. *AQP5* mediates key mechanisms of inflammation, including immune cell migration and proliferation^4,5^, activity of the renin–angiotensin–aldosterone system^6^, and the transport of water across cellular membranes^7^. Thus, *AQP5* is involved in a lot of pathophysiological properties that prevail in sepsis and its altered expression seems to represent a crucial regulatory mechanism^2^. In this context, previous studies have shown that inflammatory mediators can induce the downregulation of *AQP5* protein and mRNA expression^8,9^. In particular, there is a growing body of evidence showing that the activation of the proinflammatory NF-κB pathway attenuates *AQP5* expression^10–12^. Notably, lower *AQP5* expression attributable to the *AQP5* -1364A/C single nucleotide promoter polymorphism (SNP; rs3759129) increased survival significantly in the acute respiratory distress syndrome (ARDS)^13^ and in sepsis^14^. In addition, the AA-genotypes of the *AQP5* -1364A/C associated with greater *AQP5* expression showed increased pulmonary inflammation and an increased risk of acute kidney injury in ARDS^13,15^. Accordingly, these results suggest a protective impact of lesser *AQP5* expression in proinflammatory diseases, and mechanisms linked to an altered *AQP5* expression are of great interest.

Methylation of the cytosine residue in the sequence 5′-cytosine-phosphate-guanine-3′ (CpG) is a frequent epigenetic modification involved in the regulation of gene expression^16^ and the degree of promoter methylation can also influence *AQP5* expression^17,18^. Greater overall methylation of the *AQP5* promoter diminished reporter gene transcription, whereas demethylation by 5-azacytidine evoked greater *AQP5* expression^18^.

In the context of sepsis, there is growing evidence that epigenetic modifications can affect protein expression^19–21^; hence, *AQP5* promoter methylation might be a mechanism influencing *AQP5* expression. However, it is unknown whether *AQP5* expression under septic conditions might be epigenetically regulated by an altered promoter methylation. Accordingly, we tested the hypotheses that DNA methylation at a specific *AQP5* promoter binding site is associated (1) with altered *AQP5* expression, (2) 30-day survival of septic patients and (3) altered NF-κB binding.

## Results

Table 1 shows the characteristics upon ICU admission of the 135 study patients (78 men [58%], 57 women [42%], mean age: 57.5 yrs. ± 16 SD) with sepsis who were admitted to the intensive care unit (ICU). The 30-day survival observed was 65% (88/135) and the median duration of ICU stay was 25 days [IQR: 12–36 days]. All patients were white Germans of Caucasian ethnicity. As expected, some differences were noted in baseline characteristics between sepsis survivors and non-survivors, such as the SOFA score (p = 0.003), platelet count (p = 0.025), and bilirubin concentration (p = 0.011; Table 1). Moreover, non-survivors were more frequently mechanically ventilated at baseline (79.5%, 35/47) compared to survivors (44.3%, 39/88; p = 0.001). By contrast, no evidence for significant associations of 30-day survival was found for age (p = 0.757), sex (p = 0.855), body mass index (p = 0.128), necessity for continuous hemofiltration/dialysis (p = 0.129) and Simplified Acute Physiology Score II (p = 0.119). Moreover, there were no mortality-dependent patterns regarding infection type (p = 0.581) or comorbidities (p = 0.969).

**Table 1:** Characteristics of septic patients at baseline stratified by 30-day survival (n = 135).

	Total cohort	Sepsis survivors	Sepsis non-survivors	p-value
	n = 135	n = 88	n = 47	
**Characteristics**				
Age [years]*	57.5 (±16.0)	57.9 (±15.9)	56.9 (±16.0)	0.757
Sex, male n (%)	78 (57.8%)	50 (56.8%)	28 (59.6%)	0.855
Height [cm]*	170 (±12)	171 (±12)	170 (±12)	0.728
Weight [kg]*	80.7 (±21.5)	82.6 (±20.7)	77.2 (±22.8)	0.239
Body mass index [kg/m^2^]*	27.2 (±5.5)	27.8 (±5.3)	26.0 (±5.9)	0.128
SAPS II score*	41.8 (±18.0)	39.4 (±19.2)	44.6 (±15.9)	0.119
SOFA score*	11.4 (±5.1)	10.6 (±5.7)	12.9 (±3.4)	0.003
Procalcitonin concentration [ng/ml]^#^	3.2 (0.9–12.6)	2.8 (0.9–8.1)	3.8 (1.0–18.1)	0.386
C-reactive protein concentration [mg/dl]^#^	12.4 (5.7–20.5)	12.6 (5.3–20.5)	12.1 (6.1–19.6)	0.723
Leukocyte concentration [nl^−1^]^#^	12.8 (9.0–19.3)	12.6 (9.2–18.2)	16.2 (8.1–20.2)	0.652
Creatinine concentration [mg/mL]^#^	1.6 (1.0–2.2)	1.5 (0.9–2.0)	1.8 (1.1–2.6)	0.101
Platelet count [nl^−1^]^#^	112 (60–227)	143 (81–235)	95 (52–168)	0.025
Total bilirubin concentration [mg/dL]^#^	1.1 (0.5–2.6)	0.9 (0.4–2.0)	1.4 (0.8–3.5)	0.011
Continuous haemofiltration/dialysis, n (%)	57 (42.2%)	33 (37.5%)	24 (51,0%)	0.129
Mechanical ventilation, n (%)	74 (54.8%)	39 (44.3%)	35 (79.5%)	0.001
**Blood cultures, n (%)**				0.581
Gram-positive isolates	30 (22.2%)	18 (20.5%)	12 (25.6%)	
Gram-negative isolates	38 (28.1%)	23 (26.1%)	15 (31.9%)	
Fungal isolates	6 (4.5%)	3 (3.4%)	3 (6.4%)	
Mixed isolates	22 (16.3%)	15 (17.0%)	7 (14.9%)	
Negative blood cultures	39 (28.9%)	29 (33.0%)	10 (21.3%)	
**Site of infection, n (%)**				0.446
Pneumonia	49 (36.3%)	34 (38.6%)	15 (31.9%)	
Urinary tract infection	31 (23.0%)	22 (25.0%)	9 (19.1%)	
Abdominal infection	14 (10.4%)	6 (6.8%)	8 (17.0%)	
Skin or muscle infection	6 (4.4%)	3 (3.4%)	3 (6.4%)	
Line infection	10 (7.4%)	6 (6.8%)	4 (8.5%)	
Other/unknown origin	25 (18.5%)	17 (19.4%)	8 (17.1%)	
**Medical history, n (%)**				0.969
Cardiovascular disease	35 (25.9%)	23 (26.1%)	12 (25.5%)	
Haemato-oncologic disease	7 (5.2%)	3 (3.4%)	4 (8.5%)	
Gastrointestinal disease	34 (25.2%)	24 (27.3%)	10 (21.3%)	
Gastrointestinal cancer	16 (11.8%)	10 (11.4%)	6 (12.8%)	
Lung disease	22 (16.3%)	15 (17.0%)	7 (14.9%)	
Lung cancer	5 (3.7%)	2 (2.3%)	3 (6.4%)	
Urogenital disease	7 (5.2%)	5 (5.7%)	2 (4.3%)	
Urogenital cancer	5 (3.7%)	4 (4.5%)	1 (2.1%)	
Trauma	4 (3.0%)	2 (2.3%)	2 (4.3%)	

The data are presented as n (%) using Fisher’s exact tests, *) mean (±SD) using the Student’s t-test, or ^**#)**^ median (25^th^ to 75^th^ percentile) using the Wilcoxon-Mann-Whitney test. The following missing data were excluded from the analysis: 2 cases missing for body mass index; 6 cases missing for C-reactive protein concentration; 19 cases missing for procalcitonin concentration; 8 cases missing for leukocyte concentration; and 4 cases missing for SAPS II Score. SOFA score: Sepsis-related Organ Failure Assessment score; SAPS II score; Simplified Acute Physiology score.

**Figure 1:**
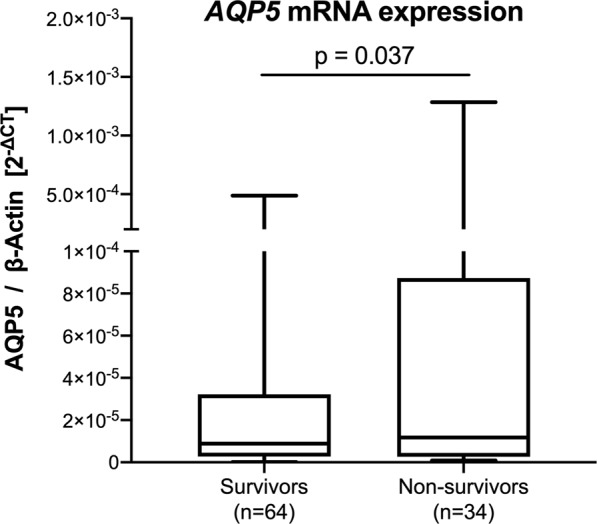
Relative *AQP5* mRNA expression of sepsis survivors and sepsis non-survivors. *AQP5* expression was normalized to β-Actin. Data obtained from the DNA of whole blood cells and presented as a box plot covering the first, second (median), third quartile, and 5th + 95th percentile. Outliers are depicted as a single dot. The p-value is estimated by the Mann-Whitney U test.

**Figure 2:**
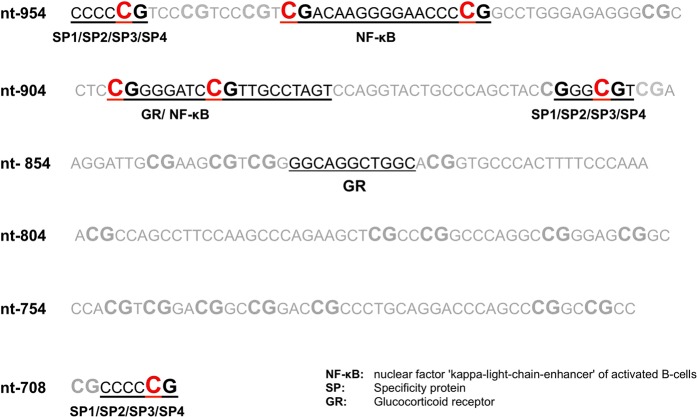
*In silico* analysis of the *AQP5* promoter region (nt-701 to nt-954) revealing binding sites for transcription. Bindings sited are shown as underlined letters. Associated transcription factors are given under each binding site. Red marked Cs represent a specific promoter position that was analyzed regarding methylation.

*AQP5* mRNA expression in the whole blood of sepsis non-survivors was significantly greater compared to non-survivors (p = 0.037, Fig. 1). *In silico* analysis exposed seven putative CpG transcription factor binding sites within the *AQP5* promoter region (nt-701 to 954): nt-701, nt-860, nt-893, nt-901, nt-922, nt-937 and nt-950 (Fig. 2). Nuclear factor NF-κB may bind to cytosine positions nt-937, nt-922, nt-901 and nt-893. Specificity protein 1/2/3/4 may bind to cytosine positions nt-950, nt-860 and nt-701, and the glucocorticoid receptor may bind to cytosine positions nt-901 and nt-893 (Fig. 2).

Accordingly, methylation analysis specific to all seven cytosine positions was performed and the data was corrected for multiple testing using the Benjamini-Hochberg method to keep the false discovery rate below 5%. Even after adjustment for multiple testing, the position nt-937 showed a statistically significant difference (p = 0.002, p_adj_ = 0.014) in methylation rate when comparing survivors (15%, IQR: 11–17) and non-survivors (17% IQR: 16–21, Fig. 3). By contrast, the other six positions (Fig. 4) and overall methylation analysis across the complete *AQP5* nt-547 to nt-1081 promoter region revealed no statistical differences between sepsis survivors (17.2%, IQR: 14.7–18.8) and non-survivors (17.9%, IQR: 15.5–20.2, p = 0.158).

**Figure 3:**
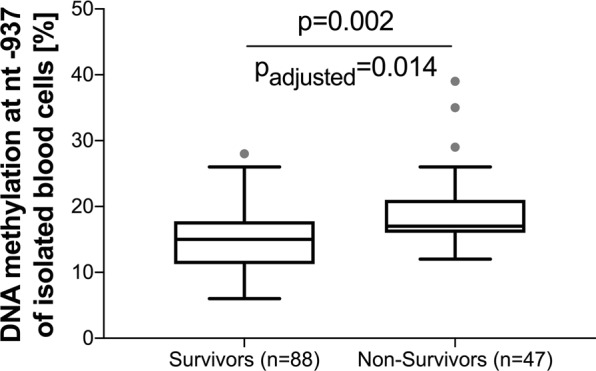
*AQP5* promoter methylation in sepsis survivors and non-survivors at position nt-937. Data obtained from the DNA of blood cells and presented as a box plot covering the first, second (median), third quartile, and 5^th^ + 95^th^ percentile. Outliers are depicted as a single dot. The unadjusted p-value is estimated by the Mann-Whitney U test; the adjusted p-value is estimated by the Benjamini-Hochberg correction for multiple testing.

**Figure 4:**
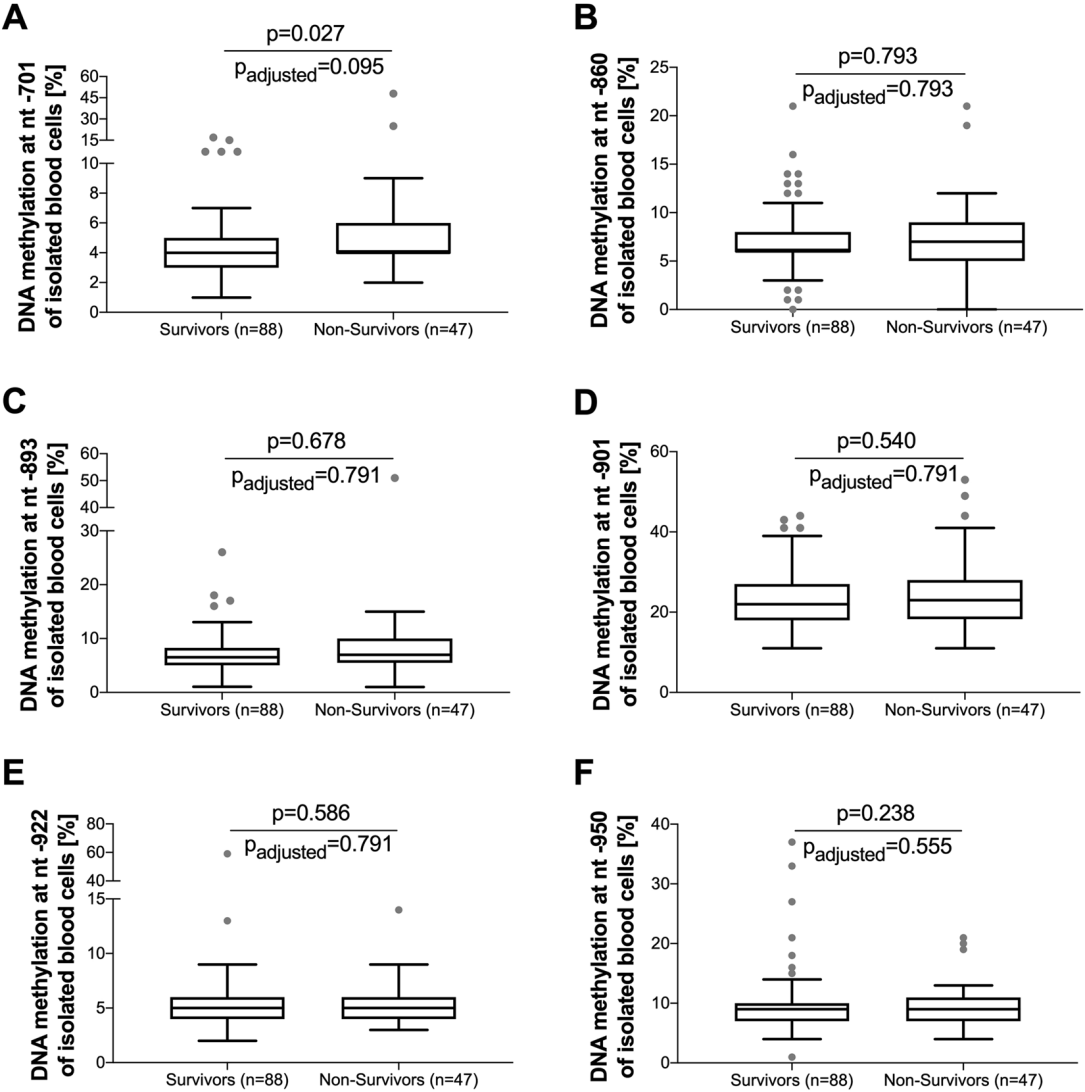
*AQP5* promoter methylation in sepsis survivors vs. non-survivors at position nt-701, nt-860, nt-893, nt-901, nt-922 and nt-950. Data obtained from the DNA of blood cells and presented as box plots covering the first, second (median), third quartile and 5th + 95th percentile. Outliers are depicted as a single dot. The unadjusted p-values are estimated by the Mann-Whitney U test; the adjusted p-values are estimated by the Bejamini-Hochberg correction for multiple testing.

**Figure 5:**
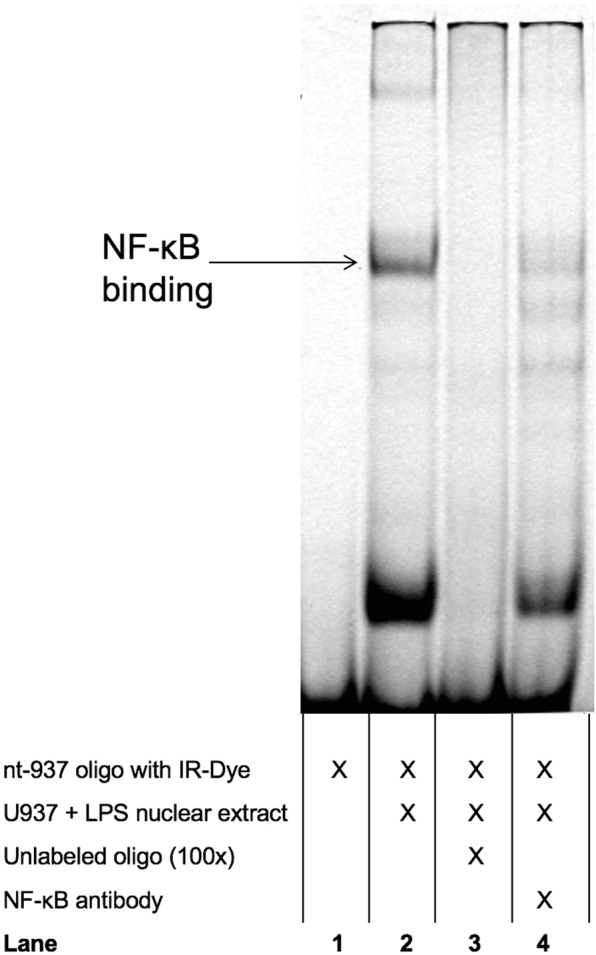
Electrophoretic Mobility Shift Assay (EMSA) was performed with oligonucleotides representing the promoter region nt-922 to nt-940, including the CpG site at nt-937. Representative blot. The addition of nuclear extracts to labelled oligonucleotides resulted in the formation of two bands (lane 2). Addition of an excess of non-labelled oligonucleotide outcompeted the specific bands (lane 3). The upper specific band is diminished with the addition of NF-κB antibody (lane 4).

To verify binding of NF-κB to the *AQP5* promoter region nt-937 an EMSA analysis was conducted covering the *AQP5* promoter region from nt-922 to nt-940. Utilizing nuclear extracts of LPS-treated U937, HeLa, and THP-1 cells resulted in formation of a specific shift, and addition of an NF-κB antibody attenuated specific binding, probably due to the affection of the DNA binding site by a NF-κB (p65) antibody (Fig. 5).

**Figure 6:**
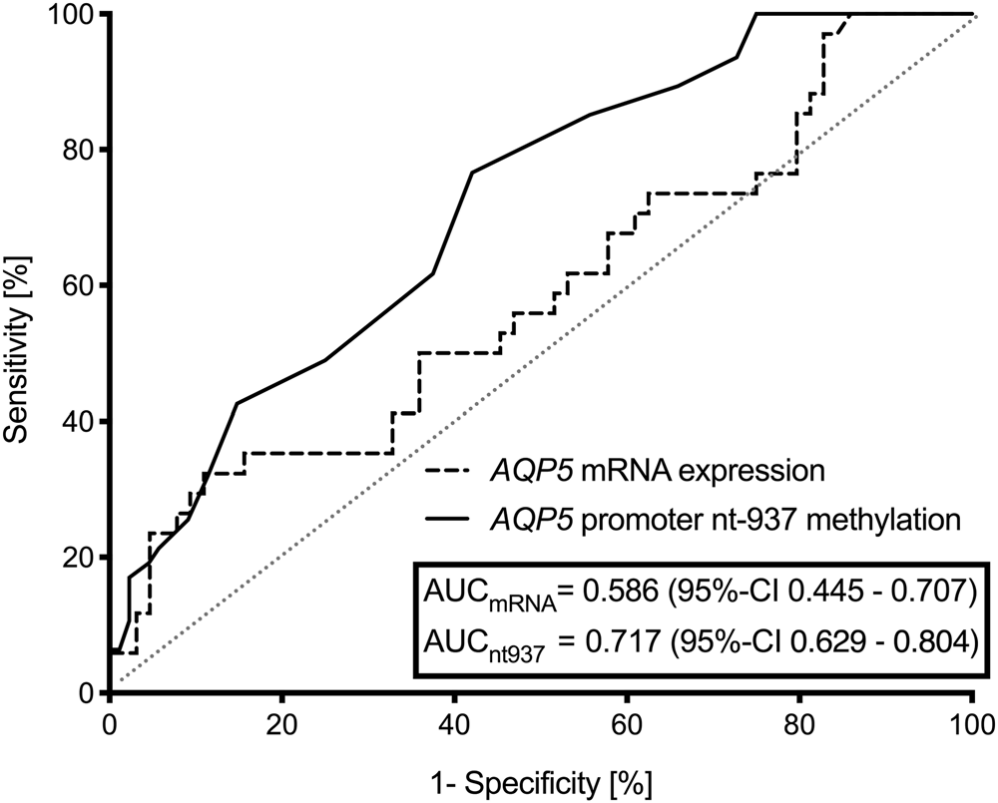
Receiver operating characteristics of *AQP5* promoter methylation at position nt-937 and *AQP5* mRNA expression in relation to 30-day mortality. The patients were stratified into patients with nt-937 methylation <16% and ≥16%. Measurements on day 1 in patients with sepsis.

**Figure 7:**
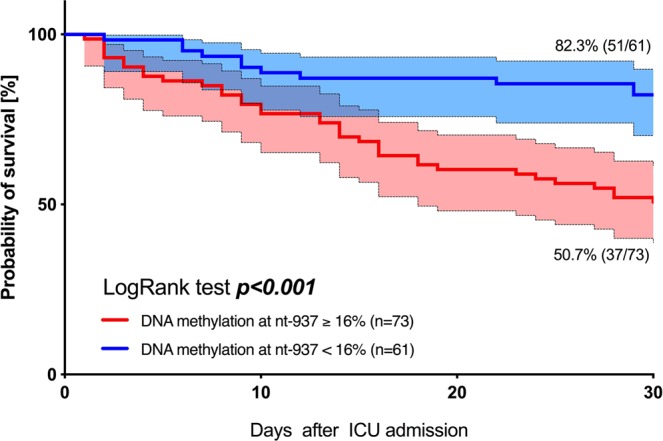
Thirty-day survival in patients with sepsis. Kaplan-Meier estimates were used to calculate probabilities of 30-day survival based on aquaporin 5 promoter methylation rates at position nt-937. The 30-day survival rate was greater in patients with a nt-937 methylation rate <16% compared to a rate ≥16%.

Next, the prognostic value of nt-937 methylation and *AQP5* mRNA expression to predict 30-day mortality was then evaluated by receiver operating characteristic curve analysis. The analysis revealed an area under the curve (AUC) of 0.717 (95% CI: 0.629–0.804; Fig. 6) for nt-937 methylation but only 0.586 (95% CI: 0.445–0.707; Fig. 6) for *AQP5* mRNA expression, suggesting a superior prognostic accuracy for nt-937 methylation. A cut-off ratio of 16% nt-937 methylated cells was determined as an optimum regarding 30-day survival using Youden’s J statistics. The related cut-off ratio of 16% showed a sensitivity of 76.6% and a specificity of 58.0% to discriminate between 30-day survivors and non-survivors. Using this cut-off, 30-day survival could be calculated as 82.3% for patients with an nt-937 *AQP5* promoter methylation rate <16% but only 50.7% for those with an nt-937 methylation rate ≥16% (p < 0.001, Fig. 7).

Multivariable Cox regression analysis referring to the methylation of nt-937 when jointly considering age, sex, SOFA score and mechanical ventilation revealed that patients with 16% or more blood cells methylated at nt-937 had a hazard ratio for death of 3.31 (95% CI 1.54–6.23, p = 0.001; Table 2). Therefore, methylation of the *APQ5* promoter site nt-937 had a significant and independent impact on 30-day survival prediction, independent of other important prognostic factors, such as the SOFA score (Table 2).

**Table 2:** Cox regression model – association between high methylation versus low methylation of the nt-937 *AQP5* promoter region regarding 30-day mortality.

	Variable	Estimates		
		HR	95% CI	p-value
Univariate	nt-937: high^$^ methylation (vs. low^§^)	3.33	(1.69–6.55)	<0.001
Multivariate	nt-937: high^$^ methylation (vs. low^§^)	3.31	(1.54–6.23)	0.002
	Age (per year)	1.00	(0.98–1.02)	0.816
	Male sex (vs. female sex)	1.00	(0.56–1.82)	0.990
	SOFA Score (per point)	1.05	(1.01–1.11)	0.043
	Mechanical ventilation	2.15	(1.10–4.21)	0.025

^$^High methylation group: ≥16% methylation of the nt-937 *AQP5* promoter region.

^§^Low methylation group: <16% methylation of the nt-937 *AQP5* promoter region.

Homer-Lemeshow statistics were as follows: κ2 = 5.50; p = 0.703.

## Discussion

This study shows that CpG methylation at the *AQP5* promoter position “nt-937” in septic patients is associated with substantially greater 30-day mortality (estimated HR 3.3) and, therefore, represents an important and independent prognostic factor. This study also provides new evidence suggesting a significant impact of NF-κB binding to the *AQP5* promoter on *AQP5* gene expression. In this regard, the binding of NF-κB seems to be diminished by CpG methylation at *AQP5* promoter position nt-937.

The *AQP5* expression in immune cells seems to impact on key mechanisms of inflammation and immune cell migration^2,22^. Notably, diseases with potentially exaggerated inflammatory responses, such as sepsis or ARDS, are associated with an attenuated *AQP5* expression, as demonstrated in different sepsis settings in human and rodent models^9,23–26^. In fact, downregulation of *AQP5* expression *may* be an adaptive reaction of the immune system that may dampen inflammation-induced harm, especially in infections with an overwhelming inflammation or a rather “dysregulated” immune response^2^.

However, regulation of *AQP5* expression varies in sepsis patients, and greater (or “less suppressed”) *AQP5* expression was accompanied by an increased 30-day mortality^13,14^. In line with these results, our present study also demonstrated a higher initial *AQP5* mRNA expression in blood cells of patients dying from sepsis compared to survivors. However, regulatory mechanisms that explain altered expression levels of *AQP5* in inflammatory diseases are still elusive.

We considered DNA methylation within the *AQP5* promoter as a possible mechanism for differential *AQP5* expression. It was shown previously that the *AQP5* gene is regulated by CpG methylation in the *AQP5* promoter region^18^. Nomura *et al*. demonstrated that a highly methylated murine cell rate was associated with a repressed *AQP5* expression^18^, while cells in a hypomethylated state expressed great levels of *AQP5*^18^. In addition, they could demonstrate that the binding of the transcription factor SP1 in cultured mouse cells was increased in the hypomethylated state and contributed to basal *AQP5* expression^18,27^.

In this study, we identified a functionally important CpG site at the *AQP5* promoter position nt-937. The latter showed an increased methylation in non-surviving septic patients and was associated with greater *AQP5* expression compared to sepsis survivors. Thus, we speculate that the binding of an “inhibitory” transcription factor to the specific promoter region around nt-937 might decrease promoter activity and subsequent gene expression. Notably, we could already demonstrate that the promoter region around this CpG site probably depicts an important promoter region and, therefore, may harbor silencer motives^6^. Strikingly, we could also confirm the binding of NF-κB to this specific *AQP5* promoter region, as the specific band was attenuated by the addition of NF-κB (p65) antibody, supporting the evidence that NF-κB signaling acts in an inhibitory fashion on regulation of AQP5 expression^10,11,28,29^. Interestingly, several studies referring to an altered *AQP5* expression in ARDS or sepsis could not find differential NF-κB or cytokine expression between survivors and non-survivors^5,13,14^. In this context, our findings may also provide a rationale that dissimilar *AQP5* expression in sepsis may be facilitated by an altered NF-κB binding ability attributable to CpG methylation rather than NF-κB protein expression. Thus, it seems appropriate to suggest that CpG methylation at the *AQP5* promoter position nt-937 attenuates the inhibitory effects of NF-κB on *AQP5* expression and may explain the relationship between inflammation and reduced *AQP5* expression. Therefore, our findings may reconcile hitherto conflicting results and provide a plausible explanation for the altered AQP5 expression in sepsis even with impact on the patients’ outcome. However, further experiments also with regard to the *AQP5* -1364 A/C single nucleotide polymorphism genotype are needed to fully elucidate and understand the potential impact of epigenetic alterations on AQP5 expression and outcome in sepsis^14,15^.

One of the important questions to be addressed in follow-up investigations is how overexpression of different subunits and the subsequent change of NF-κB dimer composition affects AQP5 promoter activity containing methylated or unmethylated CpG nt-937. In addition, assessment of the kinetics and impact of NF-κB dimer formation and their impact on AQP5 expression is warranted. Finally, it seems prudent to analyze the methylation of nt-937 in different isolated immune cells, such as neutrophils, monocytes, and lymphocytes, so as to discover potential cell-type specific differences. However, we could not demonstrate a relevant association of different blood cell counts with AQP5 promotor methylation at CpG site nt-937 (Supplementary Fig. 1). Simultaneous examination of the AQP5 promoter methylation and expression in different immune cells and at sequential time points could provide additional insight into molecular mechanisms. Apart from the mechanistic insights, our aim was to identify a potential prognostic marker for sepsis, that could easily be measured in DNA samples obtained from whole blood since DNA is a stable and abundant material and is therefore, ideal for bedside tests. Furthermore, altering methylation at the CpG-site nt-937 in the AQP5 promoter may constitute a potential therapeutic target, but robustness of this assumption should be assessed in further studies.

### Limitations

Some limitations should also be mentioned. Although all sepsis patients were treated with a rather standardized multimodal regimen, we cannot exclude the possibility that unknown and potentially confounding factors exist. While repeated measurements during the further course of sepsis may have expanded our insights, associations between promoter methylation and prognosis in this study are limited to day 1 predictions upon ICU admission. In addition, some experiments of this study were performed solely on cell lines and not in cells from organs of septic patients due to ethical and methodical concerns. Therefore, results of our cell culture experiments may not allow translation to human pathology. Furthermore, the differences in promoter methylation at nt-937 in cells of survivors and non-survivors and the methylation rate generally may appear small, since other studies have shown a very high *AQP5* promoter methylation of more than 80%^30^. However, these values were only seen in leukemia or other cancer cells, and the promoters of several genes in leukemia patients are hypermethylated^31–33^. Furthermore, all these latter cells were cultured, which is *also* linked to a greater degree of *AQP5* promoter methylation^18^. Nevertheless, other studies have indicated that methylation rate differences below 10% can be decisive for the magnitude of gene expression^34,35^. Furthermore, even on an epigenetic regulatory level, i.e. micro-RNA, gene methylation and acetylation, and histone rearrangement, in addition to interactions with other regulatory mechanisms, the regulation of *AQP5* protein expression in sepsis is likely to be very complex and certainly cannot be clarified by a single study.

## Conclusions

We detected a functionally important *AQP5* promoter CpG site (nt-937) linked to the binding of the inflammatorily acting nuclear transcription factor NF-κB, with increased methylation in sepsis non-survivors. Thus, nt-937 APQ5 promoter methylation, presumably related to NF-κB binding, is prognostically relevant in sepsis and demonstrates that epigenetic changes contribute to sepsis outcome.

## Materials and methods

### Patients

This study was reviewed and approved by the Ethics Committee of the Medical Faculty of the University of Duisburg-Essen (Essen, Germany; Protocol No. 06-3078). Written informed consent was obtained from all 135 participating patients or their guardians, according to the Declaration of Helsinki, good clinical practice guidelines and local regulatory requirements, for study inclusion between 2009 and 2014. Patients were considered eligible if they fulfilled the criteria for severe sepsis as defined by Bone *et al*.^36^ Blood samples were taken within the first 24 h after criteria for severe sepsis were met. In addition, all patients included also met the criteria of the current SEPSIS-3 definition^37^. Patients’ samples were gathered from a bio-database and some of these data had been utilized for previous studies^5,38,39^.

All patients were followed up for 30-day survival calculated on the diagnosis of sepsis. Clinical and demographic data, including the Simplified Acute Physiology Score II (SAPS II) and Sequential Organ Failure Assessment (SOFA) score, were gathered within the first 24 h after the criteria mentioned above had been met. Patients were treated using a multimodal concept, including analgesia and sedation, fluid administration, protective mechanical ventilation and hemodynamic, antibiotic and diagnostic management, as described previously^14,39^.

### Blood sample collection, preparation and storage

Blood samples were collected and processed within 30 min. The DNA and RNA were each immediately extracted from whole blood using the QIAamp^®^ DNA Blood Mini Kit (Qiagen, Hilden, Germany) or RNeasy Mini Kit (Qiagen, Hilden, Germany), respectively, according to the manufacturer’s instructions. The DNA and RNA samples were shock frozen and stored at −80 °C until analysis. Separate sample aliquots were stored and thawed later for analysis of the current hypotheses, so as to avoid multiple freezing and thawing procedures.

### Real-time polymerase chain reaction (PCR) for expression analysis

The RNA samples collected from whole blood were considered eligible for analysis when their concentration was greater than 50 ng/µl, according to nanodrop measurement (ND-1000, PEQLAB Biotechnologie GmbH, Erlangen, Germany). The RNA samples from 98 septic patients were available in acceptable quality. First-strand cDNA was synthesized from 0.5 µg of total RNA using a QuantiTect Reverse Transcription Kit (Qiagen, Hilden, Germany). The qPCR reaction was performed using GoTaq® qPCR Master Mix (Promega, Mannheim, Germany), as described previously^5^. A cDNA dilution series for *AQP5* confirmed a PCR efficiency >95%, which was comparable to the efficiency of ß-actin (data not shown). Relative *AQP5* mRNA expression was measured by two-step qPCR and expressed as 2^-∆CT^.

### Overall DNA methylation analysis by pyrosequencing

Bisulphite conversion was performed with the EZ DNA Methylation-Gold™ Kit (Zymo Research, Irvine, CA, USA), according to the manufacturer’s instructions for DNA methylation analysis. Briefly, 500 ng DNA was treated with bisulphite. Subsequently, DNA was diluted to a concentration of 10 ng/µl and pyrosequencing DNA methylation analysis was performed using ZymoTaq™ PreMix (Zymo Research, Irvine, CA, USA) with the primer sets *AQP5* (forward primers) F1 + F2 and AQP (reverse primers) R1 + R2 (Supplementary Table 1). The pyrosequencing reaction was performed using the sequencing primers *AQP5* S1 + S2 (Supplementary Table 1) with the PSQ HS 96 Gold Reagent Kits (Qiagen, Hilden, Germany) spanning the promoter region from nucleotide (nt) -1081 to nt-547.

### DNA methylation analysis of potential functionally important AQP5 promoter regions

Firstly, *in silico* analysis using Genomatix (www.genomatix.de/solutions/genomatix-software-suite.html) and Patch (www.gene-regulation.com) was performed to detect putative transcription factor binding CpG sites within the *AQP5* promoter (Fig. 2). Secondly, the *AQP5* promoter methylation at the cytosine sites with putative transcription factor binding activity (nt-701, nt-860, nt-893, nt-901, nt922, nt-937 and nt-950) were derived by pyrosequencing analysis, as described above.

### Electrophoretic mobility shift assay (EMSA)

Nuclear extracts of U937 and THP-1 cells, stimulated with LPS (1 µg/µl) for 2 h were retrieved using a Nuclear Extraction Kit (Abcam, Cambridge, UK). The DY-682 fluorescence-labelled and non-labelled oligonucleotides were used for competition analysis (MWG eurofins, Ebersberg, Germany).

All oligonucleotides were hybridized by slowly cooling down from 100 °C. The EMSA was carried out with an Odyssey EMSA buffer kit (LiCor Bioscience, Lincoln, Nebraska, USA). The probes were incubated with 5 μg nuclear extracts for 20 min at room temperature. Amounts of 100x excess of non-labelled double-stranded oligonucleotide were added for competition analysis. An amount of 2 μg of nuclear factor kappa-light-chain-enhancer of activated B-cells (NF-κB) p65 antibody (Santa Cruz Biotechnology, Dallas, Texas, USA) was preincubated for 90 min with nuclear extracts at 4 °C for super-shift analysis. The bands were visualized with an Odyssey® Imaging System (Li-Cor biosciences, Lincoln, Nebraska, USA). All experiments were performed in triplicate.

### Statistical analysis

The characteristics of patients are reported as numbers and percentages for categorical variables, means and standard deviations (±SD), or medians with interquartile ranges (25th; 75th percentile) for continuous variables, as appropriate. Categorical variables and continuous variables were compared by the chi-square or Fisher’s exact tests, Student’s t- or Wilcoxon-Mann-Whitney tests, respectively, as appropriate. All variables assessed were tested for normal distribution using the Kolmogorov-Smirnov test. Analysis of the seven different CpG sites in the *AQP5* promoter region was corrected according to the Benjamini-Hochberg procedure for multiple testing to keep the false discovery rate below 5%. Otherwise, an *a priori* alpha error p of less than 0.05 was considered statistically significant.

Predictive validity of *AQP5* mRNA expression and DNA methylation (i.e. the *AQP5* promoter region nt-937) regarding 30-day mortality was assessed with receiver operator characteristics (ROC) and corresponding results for the AUC. In a second step, ROC analysis regarding 30-day mortality was used to define an optimal cut-off value using the Youden’s index to discriminate between survivors and non-survivors. Afterwards, 30-day survival was displayed using Kaplan-Meier plots with patients stratified to the cohorts above and beyond the DNA methylation cut-off value. In addition, a log-rank test for trend was performed to describe the difference between cohorts.

Univariable and multivariable Cox regression analyses adjusted for several potential confounders were used to determine whether categorized DNA methylation of the *AQP5* promoter region nt-937 was independently associated with 30-day survival. Hazard ratios (HR) and 95% confidence intervals (CI) were calculated from the Cox regression analysis to describe the effect of covariates on the hazard.

All analyses were performed using SPSS (version 24, IBM, USA) and GraphPad Prism 8 (Graph-Pad, USA) was used for graphical presentations.

## Supplementary information

Supplementary Information

## Data Availability

The datasets generated during and/or analyzed during the current study are available from the corresponding author on reasonable request.
