# Supplementary material for: DNA methylation of a NF-κB binding site in the aquaporin 5 promoter impacts on mortality in sepsis
Source: Sci Rep. 2019 Dec 6;9:18511. doi: 10.1038/s41598-019-55051-8 (PMC6898603; doi:10.1038/s41598-019-55051-8)
Supplement: Supplementary file 1 — Supplementary Information [file 41598_2019_55051_MOESM1_ESM.docx]

**Supplementary Information**

**DNA methylation of a NF-κB binding site in the aquaporin 5 promoter impacts on mortality in sepsis**

Katharina Rump^#,1^; Matthias Unterberg^#,1^; Agnes Dahlke^1^, Hartmuth Nowak^1^; Björn Koos^1^; Lars Bergmann^1^; Winfried Siffert^2^; Simon T Schäfer^3^; Jürgen Peters^3^; Michael Adamzik^1,3^; Tim Rahmel^1^

^1^ Klinik für Anästhesiologie, Intensivmedizin und Schmerztherapie, Universitätsklinikum Knappschaftskrankenhaus Bochum, D-44892 Bochum, Germany

^2^ Institut für Pharmakogenetik, Universität Duisburg-Essen & Universitätsklinikum Essen, D-45122 Essen, Germany

^3^ Klinik für Anästhesiologie und Intensivmedizin, Universität Duisburg-Essen & Universitätsklinikum Essen, D-45122 Essen, Germany

^#^ These authors contributed equally to this work

**Supplementary Table 1:**

Oligonucleotide pairs used for pyrosequencing analysis

| **Oligonucleotide name** | **Sequence** | **Annealing temperature** |
| --- | --- | --- |
| AQP5 F1 | TAGGGTGAGGGGTTTTTAGT | 60°C |
| AQP5 R1 bio | Biotin-AATAAACACCCTACCAACCTAC | 60°C |
| AQP5 S1 | TTTTAGTAGGAAGAGGGA | 60°C |
| AQP5 F2 | GGTAGGTTGGTAAGGTGTTTGTTTTTTT | 60°C |
| AQP5 R2 bio | Biotin-ACCTTCCCCTTTTCTTCT | 60°C |
| AQP5 S2 | GGTAGGATAGTTTTGTAGGATTT | 60°C |

Oligonucleotide pairs used for qPCR - AQP5 mRNA analysis

| **Oligonucleotide name** | **Sequence** |  |
| --- | --- | --- |
| AQP5 forward | 5'-TCGGTTCAGCCCCGCTCACT-3' | |
| AQP5 reverse | 5'-GCCACACGCTCACTCAGGCT-3' | |
| Actin forward | 5'-CTGGAACGGTGAAGGTGACA-3' | |
| Actin reverse | 5'-AAGGGACTTCCTGTAACAATGCA-3' | |

Oligonucleotide pairs used for Electrophoretic Mobility Shift Assay

| **Oligonucleotide name** | **Sequence** |  |
| --- | --- | --- |
| AQP5 SE | 5’-CGTCCCGTCGACAAGGGGAACCCCGGCCTGGGAGAG-3’ | |
| AQP5 AS | 5’-CTCTCCCAGGCCGGGGTTCCCCTTGTCGACGGGACG-3’ | |

**Supplementary Figure 1:**
